# Sex-related differences in response to masseteric injections of glutamate and nerve growth factor in healthy human participants

**DOI:** 10.1038/s41598-021-93171-2

**Published:** 2021-07-06

**Authors:** Abdelrahman M. Alhilou, Akiko Shimada, Camilla I. Svensson, Peter Svensson, Malin Ernberg, Brian E. Cairns, Nikolaos Christidis

**Affiliations:** 1 Department of Restorative Dentistry, College of Dentistry, Umm Al-Qura University, Makkah Al Mukarramah, Saudi Arabia; 2 Present Address: Division of Oral Diagnostics and Rehabilitation, Department of Dental Medicine, Karolinska Institutet, and Scandinavian Center for Orofacial Neurosciences (SCON), Box 4064, SE14104 Huddinge, Sweden; 3 Department of Geriatric Dentistry, Osaka Dental University, Osaka, 573-1144 Japan; 4 Department of Physiology and Pharmacology, Center for Molecular Medicine, Karolinska Institutet, 171 76 Stockholm, Sweden; 5 Section of Orofacial Pain and Jaw Function, Department of Dentistry and Oral Health, Aarhus University, and Scandinavian Center for Orofacial Neurosciences (SCON), 5674+W3 Aarhus, Denmark; 6 Faculty of Pharmaceutical Sciences, University of British Columbia, Vancouver, BC V6T 1Z3 Canada

**Keywords:** Neuroscience, Anatomy, Biomarkers

## Abstract

The neurophysiological mechanisms underlying NGF-induced masseter muscle sensitization and sex-related differences in its effect are not well understood in humans. Therefore, this longitudinal cohort study aimed to investigate the effect of NGF injection on the density and expression of substance P, NMDA-receptors and NGF by the nerve fibers in the human masseter muscle, to correlate expression with pain characteristics, and to determine any possible sex-related differences in these effects of NGF. The magnitude of NGF-induced mechanical sensitization and pain during oral function was significantly greater in women than in men (P < 0.050). Significant positive correlations were found between nerve fiber expression of NMDA-receptors and peak pain intensity (r_s_ = 0.620, P = 0.048), and expression of NMDA-receptors by putative nociceptors and change in temporal summation pain after glutamate injection (r_s_ = 0.561, P = 0.003). In women, there was a significant inverse relationship between the degree of NGF-induced mechanical sensitization and the change in nerve fiber expression of NMDA-receptors alone (r_s_ = − 0.659, P = 0.013), and in combination with NGF (r_s_ = − 0.764, P = 0.001). In conclusion, women displayed a greater magnitude of NGF-induced mechanical sensitization that also was associated with nerve fibers expression of NMDA-receptors, when compared to men. The present findings suggest that, in women, increased peripheral NMDA-receptor expression could be associated with masseter muscle pain sensitivity.

## Introduction

Temporomandibular disorders (TMDs) are defined as a heterogeneous group of pain conditions that affect the temporomandibular joint, masticatory muscles, or both^1^. Women have been shown to be more liable to suffer from TMD than men^2^. According to epidemiological studies, as many as two out of three patients with TMD are women^3^. Masticatory muscle pain (M-TMD), including fatigue, is considered one of the most common symptoms in TMDs with greater muscle tenderness on palpation in women compared to men; moreover, it negatively affects quality of life^2,4–6^. M-TMD is thought to have a multifactorial etiology resulting from a complex interaction between biological, psychological, social and environmental factors^7,8^. Regarding the biological factors, a few previous studies have investigated neurochemical markers that may be associated with M-TMD, such as N-methyl-D-aspartate (NMDA) and serotonin type 3 (5-HT_3_) receptors, as well as nerve growth factor (NGF) and substance P (SP)^9–15^. Hence, it is important to investigate further those factors to establish how they are associated with the development and maintenance of M-TMD.

NGF plays an essential role in neuronal survival and growth of sensory neurons through the activation of the high-affinity tyrosine kinase A (TrkA) receptor and the low affinity 75 kDa neurotrophin receptor (p75^NTR^)^16^. An interaction between NGF and NMDA-receptors has been reported to occur in both the central and peripheral nervous systems of animals and humans. In rats, NGF enhances NMDA-receptor mediated currents in cultured hippocampal neurons by a mechanism that appears independent of TrkA receptor activation^17^. There is evidence showing that NGF can activate the phosphorylation of NMDA receptor 2B (NR2B) subunit by activation of Trk receptors in rat spinal cord neurons^18^. NGF-induced masseter muscle pain in rats was associated with an increased expression of NMDA-receptors by putative nociceptive afferent fibers and was attenuated by local injection of an NMDA-receptor antagonist into the masseter muscle^14^. In humans, NGF increased NMDA-receptor currents in cultured hippocampal cells from women^19^. A significant correlation between glutamate and NGF levels was observed in the cerebrospinal fluid of patients with fibromyalgia^20^. These findings together suggest that NGF could influence the excitability of nociceptive pathways through the modulation of NMDA-receptors.

Studies looking at nerve fiber density changes due to NGF or other factors are mainly reported in cutaneous tissues. Injury induced to the sciatic nerve increased the density of sensory fibers in the upper dermis of rat hind paw^21^. Another study has shown that NGF, through the activation of Trk A receptors, increased nerve fiber density in the epidermis in a model for painful neuropathy^22^. In contrast, intradermal injections of NGF, in pigs, induced hyperalgesia with no detectable changes in the density of nerve fibers^23^. Moreover, no correlation has been detected between NGF and intraepidermal nerve fiber density in patients with neuropathic conditions^24^. Still, the effect of NGF on the density of nerve fibers in human muscle is unknown.

Pain characteristics after injection of NGF or glutamate into the masseter muscle replicate common symptoms reported by patients with M-TMD^25,26^. Injection of glutamate into human masseter muscle elicited pain through the activation of peripheral NMDA-receptors^10^. However, the relationship between muscle pain induced by glutamate and the expression of NR2B subunit of NMDA-receptors and SP is still unknown. Moreover, the cellular and molecular mechanisms underlying NGF-induced sensitization of human masseter muscle and the sex-related differences in its effect are not well understood. Hence, this study aimed to investigate the effect of NGF injection on the density of nerve fibers and the expression of substance P, NR2B and NGF by the nerve fibers in the human masseter muscle, to correlate expression with pain characteristics induced by either NGF or glutamate, and to determine any possible sex-related differences in these effects of NGF. Therefore, we tested the following hypotheses: (1) Injection of NGF into the masseter muscle increases the density of nerve fibers and increases the expression of NR2B and NGF; (2) the density and expressions differ in a sex-related manner; (3) the degree of mechanical sensitization induced by NGF injection is positively correlated to the expression of NR2B and NGF; and (4) the intensity of muscle pain and extent of mechanical sensitization evoked by glutamate injection into the masseter muscle is positively correlated with the expression of NR2B.

## Methods

### Participants

It was estimated that a group size of 12 healthy men and 12 healthy women would permit the identification of a difference of 30% (± 25%) in nerve fiber expression of NR2B receptors, with a power of 0.80, and alpha set at 0.05. Further, since previous studies have indicated that significant sex-related differences in experimental pain can be identified with groups of 12 or more are investigated, no more individuals were warranted to answer our secondary aim^27,28^. However, fifteen healthy female participants and fifteen age-matched healthy male participants (mean ± SD age: 30 ± 12 years) were recruited by ads posted on the internet-page “www.forsoegsperson.dk” as well as at Aarhus University, Denmark. None of the participants had taken anti-inflammatory or analgesic medication within 24 h of the procedure. Pregnancy or facial pain, palpatory tenderness, neurological disorder, inflammatory diseases, fibromyalgia, whiplash-associated disorders, and neuropathic disorders, were considered as exclusion criteria. Female participants were not asked if they were taking oral contraceptives; however, participants using chronic medication other than contraceptive were excluded from the study. To ensure that the results are not affected by the influence of muscle tenderness on palpation, participants were screened for TMD and orofacial pain complaints using diagnostic criteria for TMD (DC/TMD)^1^. Participants received informed consent before inclusion. The local ethical committee in Aarhus County approved the experiment (approval No. 1-10-72-199-15), which followed the guidelines of the Helsinki declaration.

### Study design

The study consisted of 3 sessions at days 0, 7, and 10 and was based on a published technique for injections with glutamate and NGF^29^. On day 0, 1 M of glutamate (0.2 mL sterile solution; Skanderborg Apotek, Aarhus, Denmark) was injected on the experimental side (left side). On day 7, 0.4 mL NGF (25 μg/mL sterile solution; Skanderborg Apotek, Aarhus, Denmark) was injected on the same side. A one week wash-out period was used to avoid any possible effect of an interaction of NGF and glutamate on the muscle afferent fibers. Microbiopsies were obtained from the masseter on the control side (right side) on day 0 and on the experimental (injection) side on day 10. A control microbiopsy was taken only at the first session (day 0) to minimize harm to the participants^30^. Pain intensity at rest was recorded directly after the injections and during the following 5 min. Before (as a baseline) and 5 min after the injections, mechanical sensitivity, such as pressure pain threshold (PPT), temporal summation, as well as chewing pain and fatigue (chewing gum for 1 min) were recorded respectively from both the experimental and control sides in each session.

### Assessments of experimental pain induced characteristics

#### Pain intensity

Pain intensity after glutamate or NGF injection was recorded during 5 min on an electronic visual analog score (eVAS) ranging from 0 to 10. The lower endpoint of the eVAS was marked with “no pain” while the upper endpoint was marked “worst pain imaginable”. The highest eVAS score (peak pain) was calculated from the recorded eVAS data and used for further analysis.

#### Pressure pain threshold

PPT (KPa), the first perceived painful amount of pressure, was recorded from two sites over each masseter muscle, the injection point and a point 1 cm superior to the injection point using an electronic algometer (Somedic Sales AB, Hörby, Sweden)^31^ with a 1 cm^2^ rubber covered tip that was placed perpendicular to the skin-surface overlaying the muscle. The increase rate in pressure was 30 kPa/s. The mean of three measurements that were made over each site was used for analyses.

#### Temporal summation and chewing test

Temporal summation was assessed with 1.0 kg Palpeter (Sunstar Suisse SA, Etoy, Switzerland)^32^, by repeated applications of mechanical pressure for 1 s with a 2-s interval for ten times^33^. During temporal summation and chewing test^33^, participants were asked to rate their pain level on a 0–10 numerical rating scale (NRS) and their fatigue level on Borg’s ratings of perceived exertion (RPE) scale (6–20)^34^.

### Microbiopsies

A technique developed by Christidis et al. in 2014, to obtain enough amount of muscle tissue from an equivalent region within the muscle overall participants^35^ was used when taking microbiopsies. First, topical anesthesia (EMLA Patch, 25 mg lidocaine and 25 mg prilocaine, AstraZeneca, Södertälje, Sweden) was applied for half an hour, over the skin surface covering to the most prominent part of the masseter muscle during contraction. Then a co-axial needle within a guiding instrument (BARD TRUGUIDE; Bard Norden, Helsingborg, Sweden) was inserted with an angulation of 45 degrees, 1 cm below the zygomatic arch, along the near long axis of the muscles until the fascia was penetrated to a marked depth of 10 mm. After that, the needle was removed, while the instrument remained in place. Finally, a biopsy instrument (BARD MONOPTY) with a penetration depth of 11 mm and a diameter of 18G, was inserted through the guiding instrument to collect the masseter muscle biopsy^36^. The methodology used is based on a previous study (Christidis et al.^35^) which showed that this is a minimally invasive and painless method when compared to other biopsy methods. The needle used for skin and muscle penetration has almost the same gauge as those needles used when taking blood samples, 18 G and 21 G, respectively. Moreover, the risk of microbiopsy side effects is very rare. In the current study, no participants had postoperative inflammation; nevertheless, they were informed about the possibilities of postoperative complications before signing the informed consent and were instructed to contact the researcher directly if such complications happened.

### Immunohistochemistry and picture analysis

A blinded analysis was performed by a researcher who did not collect or code the microbiopsies. After obtaining samples, the microbiopsies were fixed over-night at 4 °C, with 4% paraformaldehyde, rinsed in phosphate-buffered saline (PBS), dehydrated, then frozen in a − 80 freezer. On the day of staining, sections with a thickness of 10 μm were incubated in normal donkey serum (ABCAM Inc, Cambridge, England, ab7475) for 1 h, and then for 24 h with primary antibodies against the specific axonal markers: (A) 1:250; anti-PGP 9.5 antibody, ABCAM Inc, Cambridge, England; ab72911; (B) 1:200; anti-NMDAR2B antibody, ABCAM Inc, Cambridge, England; ab65783; (C) 1:1000; anti-SP antibody, ABCAM Inc, Cambridge, England; ab10353; and (D) NGF 1:20; Human beta-NGF Affinity Purified Polyclonal Ab, R&D Systems Inc, 614 McKineley PL NE Minneapolis, AF-256-NA). Sections were rinsed with PBS and incubated with fluorescent secondary antibodies: (1) Alexa 488 donkey-anti-mouse, 1:700 for PGP 9.5; (2) Alexa Fluor 546 donkey anti-rabit, 1:700 for NR2B; (3) Alexa Fluor 633 donkey anti-goat, 1:700 for NGF, ThermoFisher, Burlington, ON, Canada; and (4) Alexa Fluor405 donkey anti-guinea pig, 1:700 for SP, Sigma-Aldrich, MO, USA). Removal of the primary antibodies was used as an approach to test antibody specificity. To analyze the sections as well as to capture images, a Leica TCS SPE Confocal Microscope (Leica microsystems, Wetzlar, Germany) was used.

**Figure 1:**
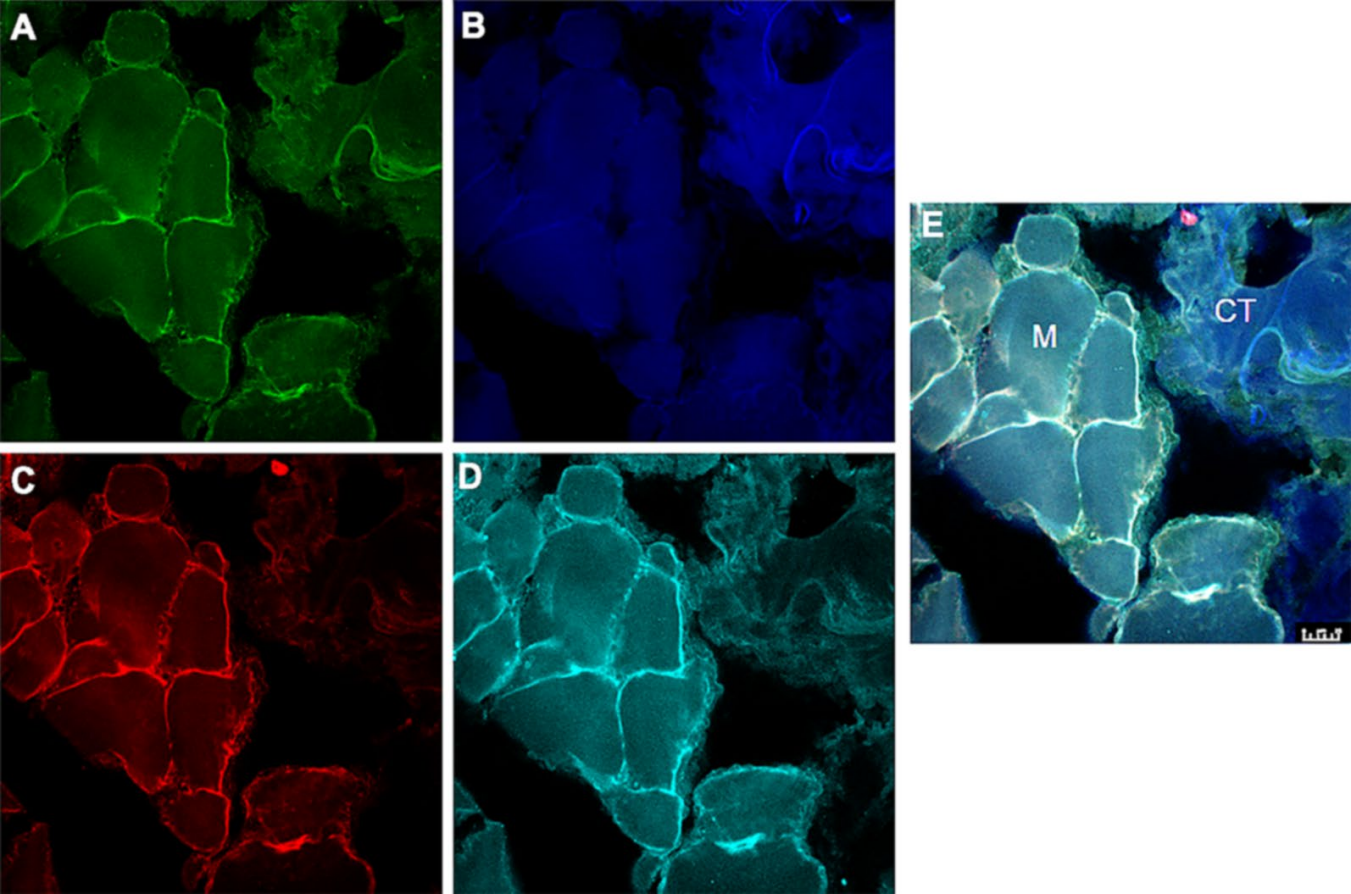
Figure displaying an example of the photomicrographs used for the analysis. The high power (×40) photomicrographs show fluorescent signaling of (**A**) PGP 9.5, (**B**) substance P, (**C**) NMDA-receptor_2B_ and (**D**) nerve growth factor (NGF) by masseter peripheral nerve fibers at day 10 (3 days post NGF-injection) from one male participant. (**E**) The composite image. *M* myocytes, *CT* connective tissue. Scale bar 25 µm.

The image processing and analysis program ImageJ (Image Processing and Analysis in Java; National Institutes of Health, USA) was used to detect and count PGP 9.5 positive nerve fibers, to calculate their area and to detect fibers (PGP 9.5) colocalization with different markers (SP, NR2B and NGF)^36^. PGP 9.5 positive nerve fibers were either associated with myocytes (semi-round or tubular, well-defined cells with multiple nuclei at the periphery) or found within connective tissue (irregular tissue containing dense or loose fibers surrounding the myocytes)^37^ (Fig. 1). A slide containing biopsies not used in immunohistochemistry was stained before analysis with Hematoxylin (HTX) to distinguish myocytes from connective tissue. Nerve fibers were considered positive if the PGP 9.5 fluorescent signals exceeded the mean background of the picture + 2 standard deviation (SD) and had a minimum length and width of 4 μm^14^. If signals were separated by 5 µm or less, however, sharing the same path and tissue were considered expressed from the same fiber. The density was calculated by normalizing the PGP 9.5 positive counts to the area of the tissue present in the images (density = number of positive fibers in a tissue divided by the total area in square millimeters of the same tissue on an image and averaged over the number of images for each participant). The expression frequency was calculated using the following formula: number of PGP 9.5 positive fibers that were colocalized with other markers divided by the total number of PGP 9.5 positive fibers in the image and averaged over the number of images for the participant. More than half of all SP expressing neurons are C-fibers and most of these fibers are nociceptors^38^. In the present study, fibers expressing SP were considered to be putative nociceptors. The immunohistochemical analysis is presented in detail in a previously published study^36^.

### Statistical analysis

SigmaPlot for Windows version 14.0 software (Systat Software Inc., San Jose, CA, USA) was used for data analysis.

#### Experimental pain

The difference in the mean peak pain intensity between glutamate and NGF was analyzed with paired t-tests. The sex differences in the peak pain after each injection were analyzed with t tests. Normalized PPT (Post-injection PPT data divided by the baseline data, multiply by 100) was analyzed with a two-way repeated-measures (2-way RM) analysis of variance (ANOVA) with factors time (Day 0, 7 and 10) and sex. The 2-way RM ANOVA was followed by post hoc comparisons with the use of the Bonferroni test. For the other parameter (temporal summation, chewing pain, and fatigue), data were not normally distributed (Shapiro–Wilk), so RM ANOVA on Ranks, factor: time (Day 0, 7 and 10), with Holm-Sidak method as a post-hoc test was used, while the Mann–Whitney U-test was used to test sex differences.

#### Immunohistochemistry

In a previous study^36^, it was shown that there was a significant difference in the expression of putative afferent sensory fibers between biopsies that contained mostly myocytes as compared to biopsies that contained mostly connective tissue. Hence, only participants whose biopsies containing the same tissue (myocytes or connective tissue) on both days were included in the analysis. The actual number of participants with tissues, regardless of its coexistence on both days, is also presented in Table 1. Pooling data from connective tissue and myocytes were intentionally avoided to prevent the risk of having an effect of one tissue on the other. For example, women on day 0 had more connective tissue than myocytes; hence pooling the data will bias the results toward expression within the connective tissue on that day.

**Table 1:** The number of participants.

	Men (n = 15)	Women (n = 15)
**Participants with same tissue both days**		
Myocytes	5	2
Connective tissue	11	13
**Day 0**		
Myocytes	8	2
Connective tissue	11	14
**Day 10**		
Myocytes	9	10
Connective tissue	13	13

The table presents the number of participants whose biopsies contained myocytes and connective tissue on both days. Moreover, it shows the number of participants whose biopsies contained myocytes and connective tissue on day 0 and day 10.

To detect significant differences and interaction between factors (day and sex) in the density and expression frequency of nerve fibers, a parametric 2-way RM ANOVA test was used and followed by post hoc comparisons with the use of the Bonferroni test. Due to the lack of samples containing myocytes in women on day 0, sex differences for myocytes are not reported.

Descriptive data are presented as mean ± standard deviation (SD) or median (IQR) depending on the distribution. The Spearman test was used to examine the correlation between the changes in parameters after glutamate injection and the expression of NR2B by nociceptive fibers, and also to test correlations between the change in parameters and the change in nerve fibers expression frequency after NGF injection. For all tests, the level of significance was set to P < 0.05.

## Results

### Pain characteristics

#### Peak pain intensity

**Figure 2:**
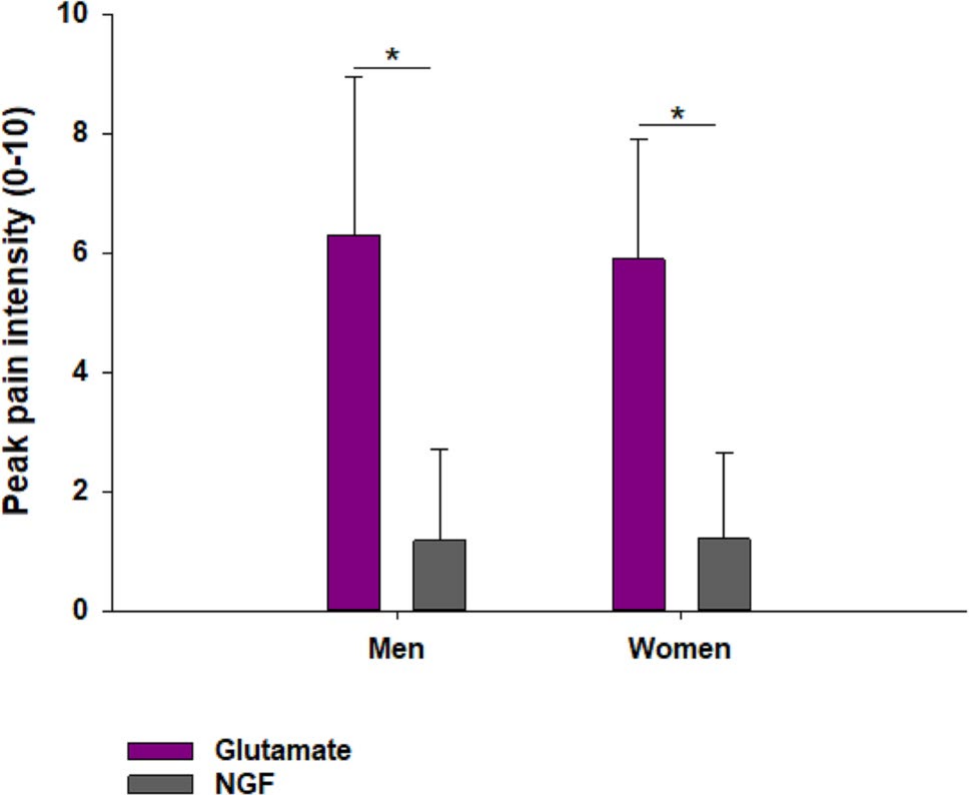
Graph illustrating the pain response to injection of glutamate and nerve growth factor. Peak pain intensities in response to injection of glutamate and nerve growth factor in 15 healthy women and 15 age-matched men. Bars represent mean and whiskers standard deviation (SD) *Significant differences (P < 0.05).

The peak pain intensity after glutamate injection was significantly greater than after NGF injection (P < 0.001), but no sex-related differences were found for either glutamate or NGF peak pain intensity (Fig. 2).

#### Pressure pain threshold

**Figure 3:**
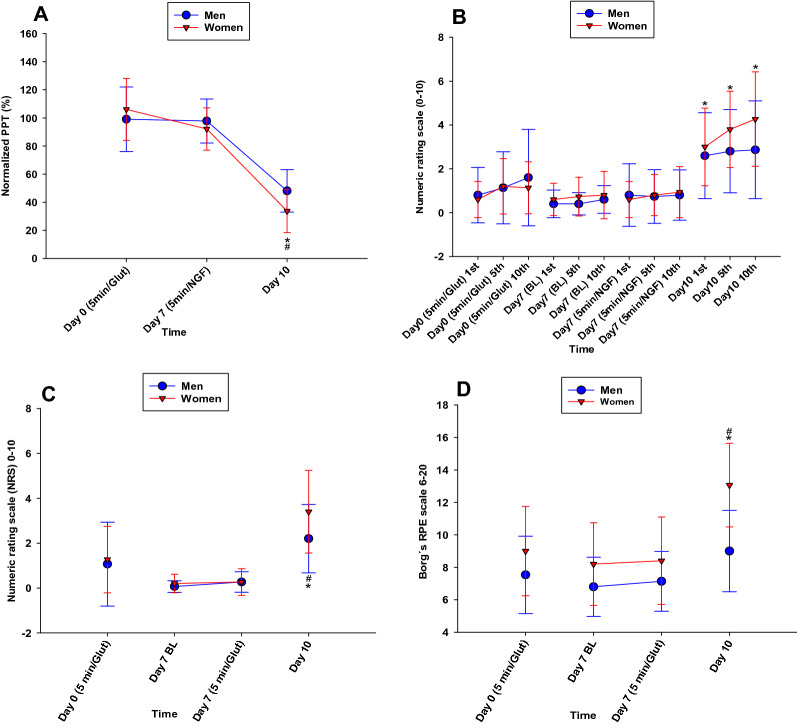
Graphs illustrating muscular sensitization by injection of glutamate and nerve growth factor. Measures of muscle sensitization assessed before (BL) and after injection of glutamate and nerve growth factor (NGF) into the masseter muscle of 15 healthy women and 15 age-matched men. (**A**) The percent change of pressure pain threshold (PPT) normalized to baseline. (**B**) Ratings of pain (NRS 0–10) for the 1st, 5th and 10th pressure stimuli applied during a temporal summation test. (**C**) Ratings of pain (NRS 0–10), and (**D**) fatigue (Borg’s RPE scale) evoked by a 1-min chewing test. All data are presented as mean (SD) for better visualization although ratings of pain and fatigue were analyzed statistically with non-parametric methods. *Significant different from other time points (P < 0.050). ^#^Significant difference between sex (P < 0.050). *BL* baseline, *5 min/Glut* five min after glutamate injection, *5 min/NGF* five min after NGF injection, *NRS* numerical rating scale, *RPE* rating of perceived exertion.

Five min after the injection of glutamate on day 0, there were no significant changes in PPT (P = 0.824) (Fig. 3A). The intramuscular injection of NGF on day 7 resulted in a significant decrease of PPT (P < 0.001) three days after injection (day 10) when compared to baseline (day 0), with a mean percentage decrease of 59.1%. PPT was also significantly lower on the experimental (left) side when compared to the control (right) side on day 10 (P < 0.001) with a mean decrease of 58.3%. The decrease in PPT on the experimental side was significantly greater in women than in men on day 10 (P = 0.015). The PPT decrease between day 0 (BL) and day 10 was 51.9 ± 15.1% in men and 66.3 ± 15.1% in women.

#### Temporal summation and chewing test

During temporal summation (on the experimental side), participants rated their pain intensity as significantly higher three days after injection of NGF (day 10) when compared both to baseline (day 0) (P < 0.001) and immediately prior to injection of NGF (day 7) (P < 0.001) Fig. 3B. There were no significant sex-related differences with respect to pain intensity produced by temporal summation at any time-point (P > 0.05). No significant changes could be detected on the control side (P > 0.05).

On the experimental side and during the chewing test, there was a significant increase in pain intensity and fatigue three days after injection of NGF (day 10) when compared to the other time points (P < 0.05) (Fig. 3C,D). Chewing induced pain and fatigue in both sexes with similar intensity at all time points, except for day 10. On day 10, women displayed a significantly higher pain intensity and fatigue score than men (P < 0.05) (Fig. 3C,D). No significant changes could be detected on the control side (P > 0.05).

### Immunohistochemistry

#### The effect of NGF on the density of nerve fibers and the expression frequency of receptors and neuropeptides

Nerve fibers associated with myocytes or nerve fibers within connective tissue revealed no significant differences in the average density of PGP 9.5 positive nerve fibers, the average density of putative afferent fibers and the expression frequency of markers (NR2B, SP, NGF) between days (0 and 10), or between sexes. The mean (SEM) density and expression in nerve fibers is presented in Tables 2 and 3. No significant interaction between factors was detected (P > 0.05).

**Table 2:** The density and expression of nerve fibers associated with myocytes.

	Day 0	Day 10	Men	Women
Density of all nerve fibers	258 (70)	339 (70)	256 (57)	342(90)
Density of fibers expressing SP	33 (10)	68 (10)	15 (4)	86 (7)
% of fibers expressing SP	10 (5)	20 (5)	8 (3)	22 (5)
% of fibers expressing NR2B	80 (6)	82 (6)	82 (4)	80 (6)
% of fibers expressing NGF	72 (8)	69 (8)	80 (8)	64 (12)
% of fibers expressing SP/NR2B	5 (4)	17 (4)	6 (3)	16 (6)
% of fibers expressing SP/NGF	10 (3)	8 (3)	7 (3)	11 (5)
% of fibers expressing NR2B/NGF	66 (8)	64 (8)	72 (8)	59 (12)
% of fibers expressing SP/NR2B/NGF	4 (3)	7 (3)	5 (2)	6 (4)

The table presents the mean (SEM) of nerve fibers density (fibers/area on mm^2^), the density of fibers expressing SP (putative afferent fibers) and the frequency of nerve fibers expressing markers alone and in combination on day 0 and 10 as well as from men and women.

**Table 3:** The density and expression of nerve fibers within connective tissue.

	Day 0	Day 10	Men	Women
Density of all nerve fibers	454 (62)	440 (62)	460 (66)	433 (61)
Density of fibers expressing SP	189 (23)	206 (23)	173 (28)	222 (26)
% of fibers expressing SP	48 (3)	49 (3)	46 (4)	51 (4)
% of fibers expressing NR2B	41 (3)	42 (3)	43 (3)	41 (3)
% of fibers expressing NGF	18 (2)	16 (2)	16 (3)	19 (2)
% of fibers expressing SP/NR2B	21 (2)	24 (2)	24 (3)	21 (3)
% of fibers expressing SP/NGF	7 (1)	6 (1)	5 (1)	8 (1)
% of fibers expressing NR2B/NGF	13 (2)	13 (2)	11 (2)	15 (2)
% of fibers expressing SP/NR2B/NGF	5 (1)	5 (1)	4 (1)	6 (1)

The table presents the mean (SEM) of nerve fibers density (fibers/area on mm^2^), the density of fibers expressing SP (putative afferent fibers) and the frequency (%) of nerve fibers expressing markers alone and in combination on day 0 and 10 as well as from men and women.

#### The correlation between the markers expression and mechanical sensitivity induced by NGF

When looking at nerve fibers associated with myocytes, data from all participants showed no significant correlation in the percentage change between days for mechanical sensitivity parameters and the percentage change in the nerve fibers expression frequency for all markers (P > 0.05).

**Figure 4:**
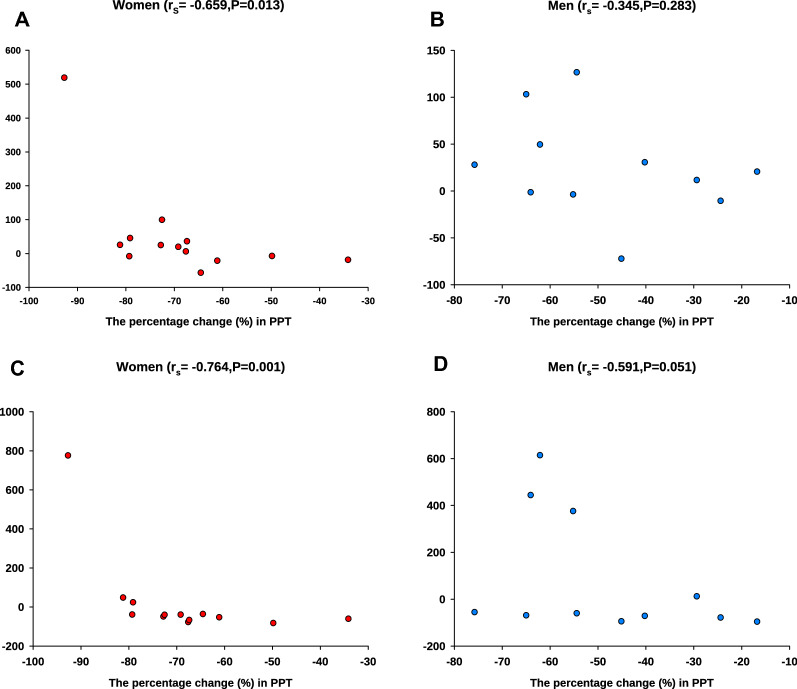
Graphs are illustrating the correlations between the percentage change in sensitization and the expression frequency of nerve fibers within connective tissue. The correlation between the percentage change (from day 0 to day 10) of pressure pain threshold (PPT) and the change (from day 0 to day 10) in the frequency of positive fibers within the connective tissue expressing (**A**,**B**) NMDA-receptor_2B_ alone and (**C**,**D**) NMDA-receptor_2B_ in combination with nerve growth factor (NGF) for women (n = 15) and age-matched men (n = 15).

Examining nerve fibers within connective tissue, data from all participants showed no significant correlation in the percentage change between days for mechanical sensitivity parameters and the percentage change in the nerve fiber expression frequency for all markers (P > 0.05). However, when data were analyzed for each sex separately, a significant negative correlation between the percentage change in the nerve fiber expression of NR2B alone or combination with NGF and the percentage change in PPT was detected in women but not in men (Fig. 4).

#### The correlation between the expression of NMDA-receptors alone or in combination with SP and sensitization or pain induced by glutamate

For nerve fibers associated with myocytes, a significant positive correlation was found between the percent nerve fiber expression of NR2B and the peak glutamate-evoked pain intensity in all participants (r_s_ = 0.620, n = 10, P = 0.048, Spearman). No significant correlations with the other experimental pain induced characteristics were found after the injection of glutamate, the Spearman’s Rank Correlation Coefficient (R_s_) and P values for PPT, chewing test and temporal summation are presented, respectively (r_s_ = − 0.313, P = 0.365; r_s_ = 0.098, P = 0.759; r_s_ = 0.246, P = 0.468). No significant correlation between the co-expression of SP/NR2B and pain induced characteristics was found (P > 0.05). It was not possible to analyze for possible sex-related differences since there were only a few samples from women at baseline containing nerve fibers associated with myocytes.

**Figure 5:**
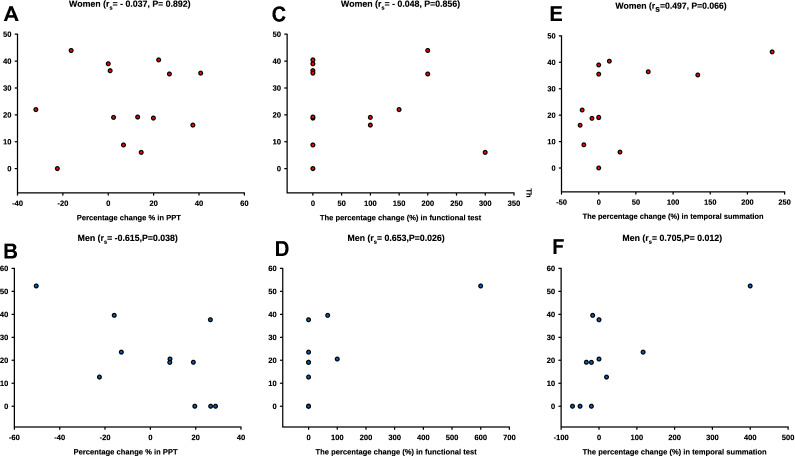
Graphs illustrating the correlations between the level of sensitization and frequency of nerve fibers co-expressing substance P and NMDA-receptor_2B_ within connective tissue_._ The correlation between the percentage change of pressure pain threshold (PPT), chewing pain and temporal summation pain (from baseline to 5 min after glutamate injection) and the frequency of nerve-fibers co-expressing substance P and NMDA-receptor_2B_ on day 0 in 15 healthy women and 15 age-matched men. (**A**,**B**) PPT in women and men, respectively. (**C**,**D**) Chewing pain (functional test) in women and men, respectively. (**E**,**F**) temporal summation pain in women and men, respectively.

Regarding nerve fibers within connective tissue, and when data were analyzed in both sexes together, a significant positive correlation was found between the percent nerve fiber co-expression of SP/NR2B and the percentage change in temporal summation pain from BL to 5 min after glutamate injection on Day 0 (r_s_ = 0.561, n = 25, P = 0.003, Spearman). No significant correlations with the other experimental characteristics (PPT, chewing test and peak pain intensity) were found after the injection of glutamate, the R_S_ and P value are presented, respectively (r_s_ = − 0.262, P = 0.204; r_s_ = 0.280, P = 0.173; r_s_ = 0.034, P = 0.867). No significant correlation between the nerve fiber expression of NR2B and pain induced characteristics (P > 0.05). When data were analyzed for each sex separately, men showed a significant positive correlation between the SP/NR2B co-expression on day 0 and the percentage change (BL and 5 min after glutamate injection; Day 0) of temporal summation pain and chewing pain, as well as a negative correlation with PPT (Fig. 5). Both in men and women, no significant correlation detected between the SP/NR2B co-expression and the peak pain intensity, the R_S_ and P value are presented respectively (r_s_ = 0.267, P = 0.416; r_s_ = − 0.154, P = 0.583). Data from women did not reveal significant correlations between the SP/NR2B co-expression and other pain characteristics (Fig. 5). No significant correlation between the nerve fibers expression of NR2B and pain induced characteristics (P > 0.05).

## Discussion

The main results of this study were (1) NGF injection into the masseter muscle did not affect the density or the expression of the muscle peripheral nerve fibers; (2) no sex-related differences were detected; (3) the decrease of PPT induced by NGF was negatively correlated with the nerve fibers expression of NR2B alone and in combination with NGF in women but not men, and (4) the pain intensity and temporal summation were positively correlated with muscle nerve fiber expression of NMDA-receptors.

In an earlier study^36^, we have shown that nerve fibers within the connective tissue and nerve fibers associated with myocytes of healthy masseter muscle express SP, NR2B and NGF differently. The results from the current study confirmed these results by showing that tissues within the masseter muscle are uniquely associated with the neurophysiological mechanisms of masseter muscle pain and sensitization. However, the following discussion will focus on the effect of NGF or glutamate injection on nerve fibers within the masseter muscle in general without differentiating tissues within the muscle.

In rats, NGF injection into the masseter muscle produced mechanical sensitization, which was greater and of longer duration in females than in males, and was associated with an increased expression of peripheral NR2B-receptors three days post-injection in both sexes, although the duration of this increased expression tended to be longer in females than in males^14^. In the present study in healthy human participants, NGF induced mechanical sensitization in both men and women, but the magnitude of this sensitization was significantly greater in women in line with previous studies^39^. NGF injections also increased pain scores during oral function in both sexes, but again the intensity of pain during these functions was significantly greater in women than in men. The NGF-induced decrease in PPT was inversely correlated with nerve fibers expression of NR2B and NR2B/NGF in women but not in men. These findings together suggest NGF induces an increased expression of NMDA-receptors by masticatory muscle nerves in women, which may, at least in part, explain the sex-related differences in this model of experimentally induced muscle pain.

Inflammation of the gastrocnemius and soleus muscle of male rats did not significantly increase the density of SP-immunoreactive fibers in the myocytes and connective tissue^40^. In human males, it has been demonstrated that intradermal injection of NGF did not increase the nerve fibers density^23^. The present study has also failed to show any significant changes in the density of muscle nerve fibers after the injection of NGF. These results together suggest that the injection of NGF does not change the density of muscle afferent fibers.

Consistent with previous reports, the current study demonstrated that NGF injection caused only low-intensity pain but significantly increased mechanical sensitivity and pain during oral function^25,39^. All previous human reports were performed on either men or women separately, so sex-related differences in function were inferred by indirect comparison^39,41^. The present study is the first, to our knowledge, to show sex differences in mechanical sensitization and pain during oral function following the administration of NGF into the human masseter muscle, which is consistent with findings in rats^14,42^.

Fatigue and pain related to masticatory muscles are common signs and symptoms of M-TMD^5^ and are suggested to be due to the accumulation of algesic substances, as well as to impaired blood flow^43–45^. In the current study, NGF produced a significant increase in pain and fatigue during chewing, which was also higher in women than in men in accordance with a recent study^46^. Hence, the combination of NGF injection with functional tests such as chewing gum might be a good model of producing reversible changes in functional pain to study analgesic approaches for TMD.

The present study showed that NGF-induced changes in PPT were inversely correlated with the nerve fiber expression of NR2B and NGF in women but not men. In rats, activation of peripheral NMDA-receptors lowers the mechanical activation of masseter muscle afferent fibers^10,47^. Local administration of the selective NMDA-receptor antagonist 5-amino-phosponovaleric acid into the masseter muscle partly reversed NGF-induced mechanical sensitization of the rat masseter muscle, which suggests an association between NMDA-receptor activity and NGF-induced sensitization^14^. These findings support the idea that the NGF-induced increased nerve fiber expression of NMDA-receptors contributes to the sex-related difference in the magnitude of mechanical sensitization induced in response to NGF-injection. Taking all these results together, one can speculate that women with a higher nerve fiber expression of NMDA-receptors may have a higher risk of developing and perhaps maintaining masseter muscle pain.

In this study, NGF injection increased temporal summation pain with similar pain intensity in men and women. This finding is consistent with a previous report that injection of NGF into the tibialis muscle facilitated temporal summation of pressure pain^48^. The present study also showed a significant positive correlation between the afferent fiber expression of NMDA-receptors and the change in temporal summation induced by glutamate injection. Temporal summation has been proposed to be a human experimental equivalent to wind-up. Both temporal summation and wind-up have been shown to depend on the activity of central nervous system NMDA-receptors^49^. Temporal summation is also dependent on the intensity of the applied stimulation. It has been previously shown that NGF injection into the rat masseter muscle decreases the mechanical threshold of afferent fibers^50^, thus potentially increasing the afferent barrage from a stimulus of fixed mechanical intensity applied to the muscle. We propose that peripheral sensitization underlies the observed NGF-induced enhancement of temporal summation in our human participants.

The present study showed that glutamate injection into the masseter muscle causes pain of high intensity in both men and women, which is consistent with previous studies^25,28^. However, many previous studies have reported greater glutamate evoked masseter muscle pain intensity in women than in men^25,27,28,51^. In a single study, which found no sex-related differences in glutamate evoked masseter muscle pain, it was suggested that this was due to the pain modulation exerted by muscle fatigue^52^. Glutamate evokes significantly greater afferent discharge in female rats, an effect that was related to estrogen-mediated increase in expression of peripheral NMDA-receptors^53^. In rats, estrogen receptors were found to be expressed in temporalis ganglion neurons, where almost 80% of these neurons also expressed NMDA receptor; moreover, the concentration of serum estrogen correlated positively with temporalis afferent discharge induced by the injection of NMDA^54^. In the present study, at baseline, we found no significant sex-related difference in the expression of NMDA-receptors by putative masseter muscle nociceptors in our human subject population. Still, a positive correlation between the peak pain intensity induced by glutamate and the expression of NMDA-receptors by nerve fibers associated with myocytes was detected at baseline. Thus, our results are consistent with the idea that increased expression of NMDA-receptors by nerve fibers is a key factor in determining pain sensitivity of the masseter muscle to injections of glutamate or NGF. However, the underlying reasons for the lack of sex-related differences in the present study are not easily identified, as several factors can contribute to the differences between sexes in rating pain. Psychophysiological factors such as attentional focusing is one of these factors^55^. Neurobiology and hormones are other factors that can contribute to the differences^53^. However, previous research found that glutamate-induced muscle pain and sensitization did not differ between women taking oral contraceptives and those who did not^28^. Moreover, there is no agreement on what phase of the menstrual cycle is associated with pain sensitivity, if present^56,57^.

### Study limitations

Due to methodological limitations, some of the biopsy samples did not contain many nerve fibers and some biopsies did not contain muscle tissue i.e., data related to the myocytes in women at day 0. This may have confounded data interpretation by decreasing the number of subjects for whom expression could be assessed and might have led to a conclusion of no significant difference between days, where that decision might have been affected by being underpowered. Another possible limitation is that SP was the only neuropeptide used to identify sensory afferent fibers, which likely underestimates the number of muscle nociceptors, because other neuropeptides, such as calcitonin gene-related peptide (CGRP), and other receptors, such as 5-HT_3_ and TrpV1, are also expressed by subgroups of muscle nociceptors. In the study conducted by Wong and co-workers in 2014, CGRP was expressed in more than 60% of trigeminal ganglion neurons that project to the masseter muscle, and it is known to be expressed by more than 70% of the trigeminal ganglion neurons in rats^58^. However, when this study was conducted, there was no CGRP antibody available for humans compatible with the PGP 9.5 and NMDA antibodies used. Finally, there was no information on oral contraceptive use; however, in most Western countries, it is not easy to recruit healthy young women who are not on oral birth control, so it is likely that the majority of women in the study were taking oral contraceptives.

## Conclusions

Injection of NGF into the masseter muscle produces a greater magnitude of mechanical sensitization in women than in men, and in women, this is significantly associated with the expression of peripheral NMDA-receptors by nerve fibers. This finding may explain part of the sex-related differences in NGF-induced muscle pain. Further, the significant correlation between nerve fiber expression of NMDA-receptors and mechanical pain sensitivity in the masseter muscle suggests that women with higher receptor expression could be at greater potential risk of developing muscle pain.
